# Employment of ex-prisoners with mental health problems, a realistic evaluation protocol

**DOI:** 10.1186/s12888-015-0553-3

**Published:** 2015-08-01

**Authors:** Ian S. Hamilton, Justine Schneider, Eddie Kane, Melanie Jordan

**Affiliations:** Doctoral researcher at the University of Nottingham, School of Sociology and Social Policy, Law and Social Sciences Building, University Park, Nottingham, NG7 2RD UK; Associate Fellow, Institute of Mental Health, Jubilee Campus, University of Nottingham Innovation Park, Triumph Road, Nottingham, NG7 2TU UK; Professor of Mental Health and Social Care, University of Nottingham, School of Sociology and Social Policy, Law and Social Sciences Building, University Park, Nottingham, NG7 2RD UK; Director, Centre for Health and Justice, Institute of Mental Health, Jubilee Campus, University of Nottingham Innovation Park, Triumph Road, Nottingham, NG7 2TU UK; Assistant Professor in Criminology, University of Nottingham, School of Sociology and Social Policy, Law and Social Sciences Building, University Park, Nottingham, NG7 2RD UK

**Keywords:** Individual placement and support, IPS, Mental health, Mentally disordered offenders, Employment, Realistic evaluation, Recidivism, Social network, Social stability

## Abstract

**Background:**

Offenders with a mental illness are routinely excluded from vocational services due to their mental health. Employment has shown to be very important in improving mental health, reducing recidivism, and connecting people to society. This study examines the effectiveness of an established intervention which is relatively untested in this population, Individual Placement and Support (IPS), to help offenders with mental health problems into competitive employment. The overall research question is whether IPS is effective in gaining and sustaining competitive employment for offenders with a Severe Mental Illness (SMI). The context is an English criminal justice setting across different populations. The study will also measure non-vocational outcomes such as recidivism, mental health and social stability.

**Methods/Design:**

A Realistic Evaluation (RE) design will address the questions “What works, for whom, and in what circumstances?” This study includes pre and post comparisons for a cohort of approximately 20 people taking part in IPS, and a similar number of controls, over a one year period. The RE also consists of interviews with practitioners and offenders in order to understand how IPS works and develops within the criminal justice system (CJS). By applying this framework the research can go from discovering whether IPS works, to how and why (or why not) IPS works. This is achieved by examining where the intervention is occurring (Context (C)), the mechanisms (M) that create particular behaviours, and how the outcomes (O) from the intervention all come together (CMOs). Employment outcomes will also be examined for all participants.

**Discussion:**

By applying RE the research will permit inferences to be drawn about how and why (or why not) IPS works, by examining context, mechanisms and outcomes. IPS has never been implemented within the CJS in the United Kingdom. As a result, this evaluative research will not only provide a novel insight into the core research areas, but also how the intervention can be improved for others in the future.

## Background

Mental illness could be defined as “health conditions that are characterised by alterations in thinking, mood, or behavior (or some combination thereof) associated with distress and/or impaired functioning” [1], p. 5. Only 7.3 % of people with severe and enduring mental health problems are in employment [2]. This is in stark contrast to the 73 % of those employed among the general population within the United Kingdom (UK) [3]. Furthermore, the gap between employment in the general population and people with any mental illness within England is 37.1 % [4]. In other countries, too, people with a mental illness are also up to 50 % more likely to be unemployed than those with other disabilities [5].

Employment figures are even worse amongst those within the Criminal Justice System (CJS). The Ministry of Justice (MoJ) has previously aimed to increase work opportunities within prisons, however this does not seem to be improving employment and/or opportunities to gain employment upon release [6]. For example, Niven and Stewart found that 70 % of working-age prisoners in England and Wales were found to have no form of employment and/or education upon release [7]. Even prior to entering prison, 67 % of prisoners have been shown to be unemployed [8]. Furthermore, in 2013/14, only 25 % of prisoners entered employment when released from prison [9] indicating that offenders are failing to attain the benefits-such as social engagement, economic stability and independence, which employment can offer them on their release.

The mental health of prisoners has been consistently worse by comparison with the general population [10, 11]. For example, an eminent study carried out within England and Wales by Singleton, Meltzer and Gatward found that some 90 % of prisoners were suffering from a mental health condition whilst more than 70 % had two or more mental health disorders [12]. Even though this research is more than a decade old, it was conducted within a population which has more than doubled within the last 20 years [13] and as a result now has one of the highest rates of imprisonment in Western Europe [14]. Recent figures indicate that 62 % of male and 57 % of female prisoners who are serving a sentence have a diagnosable personality disorder [15]. The proportion of males and females in prison who suffer from depression and anxiety is 23 % and 49 % respectively, while 25 % of female prisoners and 15 % of male prisoners also report symptoms indicative of psychosis [16]. This high prevalence of mental illness amongst prisoners is also reflected in the 23,183 reported self-harm incidents in the 12 month period ending December 2013 [17].

Consequently, it is reasonable to infer that there are more people in prison with mental health problems at present than ever before [18]. Indeed, research has consistently demonstrated prison environments to have a large number of prisoners with unmet mental health treatment needs [18–21].

These findings are a significant concern as employment has shown both to improve an individual’s mental health and quality of life [22], and to reduce re-offending by up to 50 % [8, 23, 24]. Research in the UK also indicates not only that the vast majority of offenders want to stop offending, but that they see employment as key to achieving this [25].

However, people with a mental illness and criminal justice involvement are often routinely excluded from resettlement vocational services due to their illness and thus are perceived to be ‘not ready for work’ [26]. A criminal history is also widely presumed to be a significant barrier due to the employers’ screening procedures for applicants [27]. The social stigma of a mental illness together with the criminal record held by individuals have been described as a “double jeopardy” [22], p. 1. The adverse effect of these barriers is further evidenced in that clients with a SMI and criminal justice involvement have delayed entry onto vocational services compared to those who do not have criminal justice involvement [28].

### The intervention: IPS

The IPS model consists of a set of core principles that have been extensively examined using continuous feedback from all those involved within the field; real world research data has also been utilised as a means to bring together service ideas, outcomes, and to best adapt to the needs of clients who have a SMI [29–33]. As a result, the IPS model has been extensively studied and developed internationally [34] and is now the leading evidence-based approach to supported employment for people who have a SMI [35]. There are currently eight features to the IPS model which are fairly self-explanatory, as follows [36]:

- Competitive Employment is the Primary Goal
- Zero Exclusion: Eligibility Based on Client Choice
- Integration of Employment & Mental Health Services
- Attention to Client Preferences
- Personalised Benefits Counselling
- Rapid Job Search
- Systematic Job Development
- Time-Unlimited & Individualised Support

IPS has shown success rates in achieving competitive employment to be as high as 61 % using its ‘place-train’ approach, as opposed to 23 % using a common ‘train-place’ alternative method [36]. Overall, the substantial research within the literature suggests that around 60 % of those who take part in IPS gain competitive employment, with around 50 % becoming steady workers. Furthermore, the benefits of IPS also extend to improvements in the mental health of an individual following sustained employment [37, 38].

IPS has never been implemented within an offending population in the UK, and only one similar ongoing study within the United States (US) exists, so little is known about its efficacy within a criminal justice setting. Consequently, the current project is highly distinctive. Firstly, it will examine the impact of IPS within an offending population for the first time in the UK. Secondly, it is innovative in that it will examine the impact IPS has on an individual’s mental health, social stability, and recidivism within one inquiry, using both quantitative and qualitative techniques. Thirdly, the RE approach enables the study to go beyond hypothesis-testing to elaborate causal explanations for what works for whom.

### Recruitment

This research was initially designed for delivery in Her Majesty’s Prison Service (HMPS) across three prisons. However, in order to maximise referrals onto IPS and the current study participants will be recruited from a total of seven prisons and from offenders serving community orders. Community referrals onto IPS will be taken via Integrated Offender Management (IOM). The IOM is part of a multidisciplinary team that looks after ex­offenders in the community and includes, for example, the National Probation Service (NPS), Police, and Drug and Alcohol Teams.

### Research questions

1. How is the IPS intervention working in practice? What exactly does the IPS methodology intervention consist of to be able to continue and work effectively within a criminal justice setting?
2. Is the IPS intervention successful in achieving competitive employment outcomes for ex-prisoners with mental health problems?
3. What is the effect of the IPS intervention on the mental health of ex-prisoners who have a mental illness?
4. What is the effect of the IPS intervention on the social stability of ex-prisoners who have a mental illness?
5. What is the effect of the IPS intervention on the re­offending rates of ex-prisoners who have a mental illness?
6. As a result of the above findings, how does IPS need to be adapted, if at all, to achieve the best possible outcomes in a criminal justice setting?

### Principal inclusion criteria

- Aged between 18 and 65
- Able to give informed consent
- Offered employment support through the IPS service
- Within 3 months of release date if in prison, if already released, within 6 months of release date
- Have a SMI

SMI is defined as meeting one or more of the following criteria:

- Been treated by a psychiatrist in the last 12 months
- Recently been treated by a general practitioner for chronic mental health problems and/or substance misuse difficulties
- Been in hospital in the last 2 years due to mental health problems
- Currently on the caseload of their community mental health and/or substance misuse team
- Being treated by a mental health prison in­reach team and/or on a drug intervention programme
- Being treated in the community by a primary mental health team

Staff participants recruited in prison and in the community:

- To be eligible for the current study participants must be able to give informed consent and be either involved in IPS in some form or in the overall care of participants.

### Principal exclusion criteria

Offender participants recruited from prison and the community:

- Due to the nature of the intervention, those who might not adequately understand verbal explanations or written information given in English.

### Primary and secondary outcome measures

The primary outcome measure for the study is:

Employment gained in a competitive market through the IPS intervention (yes/no).

The secondary outcome measures for the study are:

Mental health, motivation to work, social stability, admissions to a psychiatric hospital, and offending behaviour

This will be assessed at 6 and 12 month intervals. The following information will also be collected where relevant:

- Time (days) until first competitive employment
- Job tenure of competitive employment (hours/days/weeks/months worked from start of IPS)
- Job stability (working hours/contract gained)
- Income from employment

Leaving the study for any reason will be measured to assess any bias in the results.

Participant medical records­ hospitalisations, and the reasons for these, will be sought to fully understand the primary and secondary outcomes. Previous hospitalisations predict and increase the chance of future hospitalisation [39] and therefore may interrupt employment or the potential to gain employment during the study.

This medical information will also be very important when examining the mental health and motivation to work of participants throughout the study. By understanding if the participant has had any very recent previous and current hospitalisations, and why, the researcher can control for how this may mediate time until employment, participant dropouts, job tenure, and hours worked.

Information will also be sought from the participants’ prison records. Prison records would reveal any history of offending, index offence information, and the duration of sentence/community order. This information will also be important when examining any barriers a participant may have had when seeking competitive employment.

## Methods/Design

### Sample size and power

This is a novel piece of research examining how IPS works and develops within a criminal justice setting by means of a Realistic Evaluation (RE). It will yield valuable information for future studies, including the rates of recruitment and retention of participants, as well as the variation in the outcome measures, which is needed to undertake sample size calculations. The current sample size has been determined by the time and resources available: 20 (IPS) and 20 (No IPS) will have a power value of .60 and a critical z score = 1.645 [40] 1.

### Offender interviews

Previous research indicates that the employment rate of offenders released from prison is 25 % [9]. The IPS literature consistently demonstrates that participation in IPS doubles the percentage of people in paid employment [41]. However, this excludes offenders who, for reasons cited earlier, face additional obstacles to employment. Therefore, in consideration of previous IPS research and the experience of the research team, an estimated hypothesis for the primary outcome is that 40 % of those on IPS will work at least one day in paid employment compared with 25 % for those who do not take part in IPS: at least eight of the intervention group of 20 will obtain work, as compared to five of the control group. A 15 % net increase in the competitive employment rate is both clinically and socially meaningful as not only would this reduce the range of costs to society that are associated with this population, but according to previous findings employment can have a positive impact on each individual’s life satisfaction and their mental health, especially if this is sustained [37, 38].

As there is no randomisation with regards to who takes part in IPS and who does not take part in IPS, motivation to gain employment will be measured, making it possible to control for the effect of differences in motivation on outcomes using logistic regression.

Given that the majority of recruitment and consent will be taken within prison the research team estimate that most (80 %) of those approached to participate in the research will take part. Furthermore, as not everyone who is approached will take part in the research, even though they may take part in IPS, it is believed that 36 people within each group will need to be approached.

### Staff interviews

A total of around 20 staff participants will be recruited in order to gain a thorough insight and range of perspectives into how the intervention works, develops, is delivered, and fits into the CJS. Only National Offender Management Service (NOMS) staff involved in some way with the intervention and/or overall involvement with participants will be interviewed. Estimated N = 10.

Other key stakeholders and agencies involved in the overall care of participants and/or the delivery of IPS, such as employment specialists, will be interviewed once or twice only to gain an understanding of their perspective of the intervention. Interviews with such staff will occur with an estimated N = 10.

### Data collection

#### Validated measures

All of the measures seen in Table 1 are highly validated and will assess the mental health, social stability, including social network, and the motivation of each participant. Each measure is self-administered, however, the researcher will help each participant where necessary. The TAG is already routinely present on each client’s file and will be completed twice by staff who know the participant well. Each set of questionnaires will take around 30 minutes to complete.

**Table 1:** Data collection measures and time frame for offender participants

*Measure*	*Baseline*	*6 months*	*12 months*
Threshold Assessment Grid (TAG) [42]		*√*	*√*
General Health Questionnaire-12 (GHQ-12) [43, 44]	*√*	*√*	*√*
Brief Symptom Inventory-18 (BSI-18) [45, 46]	*√*	*√*	*√*
Manchester Short Assessment of Quality of Life (MANSA) [47]	*√*	*√*	*√*
Social Network Analysis (SNA) [48]	*√*		*√*
Motivation and Readiness to Work scale (MTW) [49]	*√*	*√*	*√*
Reconviction Analysis			
Semi-structured Interview	*√*	*√*	*√*

#### Semi-structured interviews

Semi-structured interviews will last between 30 to 60 minutes, a total of less than 90 minutes per offender. The researcher will follow a topic guide (available from the authors on request). It is also important to note that participants may be recalled to prison for a number of reasons other than reoffending, therefore, this will be taken into account and questions and/or prompts within this topic guide will be asked as appropriate.

Interviews will be carried out to gain an understanding of the individuals’ motivations and expectations of the IPS service and to understand how the intervention is working. Interviews will also generate data concerning the impact of IPS and/or any employment gained on the core areas under investigation- mental health, social stability, and recidivism.

All participants will initially be asked questions about their offending behaviour. Participants who have reoffended will be given an opportunity to discuss their offending behaviour and the reasons for it, within the semi-structured interviews. Those who do not reoffend will be asked questions about what they believe has helped them to desist from criminal behaviour.

The initial and subsequent interviews will take place in prison and then in the community, respectively, however, this may vary depending on referral and participant location at time of interview.

### Reconviction analysis

A sample of similar size matched on criteria that influence reoffending, will be compared with the study participants to determine the probability of reconviction with and without IPS involvement. This ‘reconviction analysis’ will also involve comparisons between the two groups on new index offence, sentence length, and overall circumstances.

### Key stakeholders and observations

Semi-structured interviews will take place with employment personnel on two occasions, once when more than five clients have built up on their caseload and once at the end of the project. Interviews will be conducted with all three employment specialists. This will help to understand their role, decisions they make, and the possible day to day barriers that they are faced with to gain competitive employment for clients.

The researcher will also need to be in contact with employment specialists to understand what their role with this new population involves. Observations will occur with each employment specialist for a three to four week period, two to three days per week. The researcher will write up notes after each day observing.

Semi-structured interviews with other key stakeholders involved in the intervention and/or the overall care of participants will be taken at the six and 12 month points where possible. Interviews with employment personnel and other key stakeholders will also both provide an overall context and ‘logic of the stakeholder’ [50]. That is, an understanding of how the service begun, has developed, where it is now, and how it may need to be adapted.

### Fidelity

To permit systematic evaluation of IPS interventions it was essential that a method be developed for assessing adherence to the IPS principles as well as differentiating from programmes that stray from the model. A fidelity scale does just this and could be described as “a psychometrically sound method for determining the degree to which a specific programme meets the standards for a programme model” [51], p. 384. Therefore, a key component in the examination of the IPS intervention and a measure that also provides a detailed operational description and critical ingredients of the IPS model is the 25-item IPS Fidelity Scale (IPS-25) [52].

The IPS intervention has now been running for several months, therefore, a self-assessment fidelity review for the service is due to take place in April 2015. This will be followed by an external review six months after the research begins. The Centre of Mental Health, London, and Shropshire County Council will be organising this process. These fidelity scores can then be integrated into the research analysis when examining employment outcomes through providing adherence to the IPS model during the development of IPS, and when the research data was being collected.

### Analysis

As the research design is RE; both the qualitative and quantitative methods will be applied to the data prior to final interpretations to understand what works, for whom and under what circumstances.

### Rigour and reliability

RE is unique from other research methods as it is able to isolate mechanisms of change within an intervention [50]. Different stakeholders will have diverse information, understandings, and divisions of expertise about how IPS has and may work. RE ensures that the correct questions are asked to the right ‘experts’ in each area under examination. This can then allow for an examination of the mechanism (s) which create new and/or continuous behaviour (s) concerning the research areas.

It is anticipated that by examining employment outcomes and the impact being involved in IPS and/or employment has and had on a client’s overall mental health, re­offending, and social stability, in comparison to those not on IPS, how to best adapt IPS within the justice system can be understood. By examining individual circumstances, mechanisms, and the context (CMOs), as well as all data collected from both ‘positive’ and ‘negative’ cases regarding who gains employment, ways in which the intervention can be improved within different contexts can then be proposed.

### Qualitative data

Interviews with clients and key stakeholders will create text that will be analysed through Thematic Analysis [53]. Themes will be recorded and refined through a systematic process using NVivo software. The researcher will identify codes in the interview material. Codes are the basic element of the data that illustrate information that is of interest to the analyst [54]. An initial list of codes will be compiled and then organised into overarching themes. A thematic summary once created can be refined through further interviews and feedback to categorise themes at each stage of outcomes produced [55].

Interviews and observations with employment personnel will be developed into themes of how IPS has developed and/or been modified within the CJS. Interviews and observations with employment personnel will also generate information into what works, and what doesn’t work, with what particular population and client.

### Quantitative data

To determine whether there was a significant difference between those who took part in IPS and those who did not, concerning whether competitive employment was gained, a Chi-square analysis will be carried out at the six and 12 month intervals. Similarly, a logistic regression will also be carried out at these intervals in order to control for a participants’ motivation to work and assess covariates (e.g. age, gender, criminality) between both groups. Motivation to work will be controlled for during all comparisons, as the decision to seek employment is a personal choice.

Questionnaires pre and post IPS will be analysed using statistical independent sample t­tests, using a Statistical Package for the Social Science (SPSS) software, to determine whether overall significant improvements exist within mental health and social stability between those who did, and those who did not take part in IPS. A case by case examination of client yes/no responses in interviews, social stability measures (job contract and income, accommodation, social network etc.), and structured aspects within social stability questionnaires will be examined in conjunction with all other data, such as themes gained from interviews, to supplement all research questions and to make improvements and recommendations concerning IPS. Unemployment rates, jobs created data, fidelity to the IPS model and any changes in crime rates for particular areas will also be taken into account when examining results.

Descriptive data will be produced to meet the additional secondary outcomes; employment outcomes achieved. All other structured information from the relevant validated measures will be examined case by case for patterns of change amongst those who were part of IPS in comparison with those who were not part of IPS. Appropriate statistical analyses will account for any attrition variation between the IPS and no IPS control condition across the three data collection points. Missing data will also be excluded using the appropriate statistical techniques.

By examining Police National Computer (PNC) data the proven one year re­offending rates of individuals who took part in IPS will be made clear. Specifically, whether any offence has been committed and whereby a conviction, caution, warning or reprimand was received within the one year follow up and six month waiting period [56]. This will also give an indication of the severity and regularity of reoffending in comparison to each individual’s own previous offending behaviour.

To generate stronger findings that go beyond whether an individual offends or not and how this compares to their previous offence history it is important to determine what would have happened to the participants on IPS should they not have taken part, an estimate is needed. This estimate is known as the ‘counterfactual’ [57] and will enable a comparison in reoffending rates of those who took part in IPS with those who did not. Making this comparison ensures that the only major difference in characteristics between both groups is the IPS intervention. In order to isolate the effects of IPS in this way those on IPS will be matched on key factors which influence reoffending with a comparator group (No IPS). A logistic regression, Propensity Scored Matching (PSM), will be utilised to carry out this comparison.

### Ethics and consent

Ethical approval for this study has been obtained from the National Health Service (NHS) Health Research Authority, reference number 15/NE/0049, and the NOMS ethics committee.

As this study will generate sensitive and personal data consideration of ethics and participant confidence is essential. Interviews will all be conducted in a sensitive manner. During interviews the participants’ verbal and non-verbal behaviour will be monitored to ensure that they are comfortable and able to continue. Participation is completely voluntary and participants may refuse to complete the study at any time or refuse to answer any of the questions for any reasons. Should the researcher feel that an individual is unstable, distressed and/or experiencing difficulties, or presents suicidal ideation, the interview will be stopped and the appropriate professionals contacted.

The researcher will also ensure that all activities and analyses are conducted to the highest ethical standards, adhering to relevant guidance. Participation in the research will be entirely voluntary and on the basis of written informed consent. Written informed consent for participation in the study will be obtained from each participant. Information sheets will also be administered to all those who may be present during observations with employment personnel in order to allow the individual (s) to opt out. Written informed consent will be obtained for participation prior to each interview and set of questionnaires administered. Information sheets and consent forms will be jargon free and created to account for diverse levels of literacy: Easy Read, for those that require such a format.

The information sheet and consent form will also ask consent for access to medical and criminal client information for the purpose of the research only. No promises of employment will be made to participants, which will also be made clear on the information sheet. Participants are not dependent on the relationship with the researcher and consequently their help to find work, if they are receiving any, will not change at any point should they withdraw or when the study ends.

Financial payments will be offered to participants for their time throughout the study. The exact details regarding how payments will be provided and when will be indicated on a separate form. This will be administered to participants when they have decided that they would like to take part in the study.

## Discussion

The study will be carried out across a wide geographical area. A total of eight sites, seven prisons and one probation area are eligible to provide referrals to the study, including both people who do and do not want help to find competitive employment via the IPS service. This will therefore involve conducting research within the demands of a criminal justice setting, with a population many of whom have chaotic lifestyles.

## Abbreviations

MoJ: Ministry of Justice
SMI: Severe Mental Illness
CJS: Criminal Justice System
IPS: Individual Placement and Support
IOM: Integrated Offender Management
NPS: National Probation Service
HMPS: Her Majesty’s Prison Service
NOMS: National Offender Management Service
PNC: Police National Computer
TAG: Threshold Assessment Grid
GHQ: General Health Questionnaire
BSI: Brief Symptom Inventory
MANSA: Manchester Short Assessment of Quality of Life
MTW: Motivation and Readiness to Work
SNA: Social Network Analysis
RE: Realistic Evaluation
CMOs: Contexts, Mechanisms and Outcomes
NHS: National Health Service
IPS-25: IPS Fidelity Scale
PSM: Propensity Scored Matching
UoN: University of Nottingham
US: United States
UK: United Kingdom
SPSS: Statistical Package for the Social Science

## Footnotes

1 The primary outcome measure for the current study is more than or equal to one day worked in paid employment. As a result of this binary measure, a Chi-square analysis is required. In line with the estimation and previous research detailed above proportion two, participants not on IPS, equates to 0.33 (25/75), and proportion one, participants on IPS, equates to 0.64 (39/61). Thus, a full study sample size of 34 (IPS) compared against a sample size of 34 (No IPS) at 6 and 12 month intervals, concerning more than or equal to one day worked in paid employment, would have the power (.80) and critical z score = 1.645 [40].
